# Strychnine as an Evacuant

**Published:** 1904-10-01

**Authors:** 


### STRYCHNINE AS AN EVACUANT.

The normal and regular evacuation of the intestinal canal is dependent on two principal factors, namely a sufficiently moist state of the bowel contents and efficient peristaltic action. Dr. G. E. Pettey[1] urges that constipation is more usually to be attributed to failure of peristalsis than to dryness of the fæces, for the contents are as a rule fluid when they first enter the large intestine. Treatment should therefore be directed towards increasing the muscular activity of the bowel rather than to causing an excessive secretion of fluid in the intestine ; and the best drug to use for the purpose is strychnine. The dose required will of course vary in different cases, but will lie somewhere between $\frac{1}{8}$ and $\frac{1}{30}$ grain given at intervals of two or three hours, until four to six such doses have been given.

[1] Merck's Archives, Sept. 1904.

